# Socio-demographic determinants of unmet need for family planning among married women in Pakistan

**DOI:** 10.1186/s12889-019-7487-5

**Published:** 2019-09-05

**Authors:** Muhammad Farhan Asif, Zahid Pervaiz

**Affiliations:** Department of Economics National College of Business Administration and Economics 40-E/1 Gulberg III, Lahore, Pakistan

**Keywords:** Family planning, Unmet need for family planning, Unmet need for spacing, Unmet need for limiting, Married women, Pakistan demographic and health survey

## Abstract

**Background:**

Family planning is considered as an effective tool to control population and to bring improvement in maternal and child health. The Government of Pakistan has been continuously struggling to improve the availability of family planning services. However, like many other developing countries of the world, unmet need for family planning still exists in the country. According to Pakistan Demographic and Health Survey 2012–13, the prevalence of unmet need for family planning is 21% in the country. The objective of this study is to investigate the determinants of unmet need for family planning among married women in Pakistan.

**Methods:**

Secondary data of Pakistan Demographic and Health Survey 2012–13 has been used to analyze the determinants of unmet need for family planning through Binary and Multinomial Logistic regressions.

**Results:**

Outcomes of the study show that the likelihood of unmet need for family planning among married women in Pakistan goes on to decrease with an increase in their age and education. The likelihood of unmet need for family planning decreases with the increase in wealth status of women’s household, number of living children and husband’s education. Similarly, the women residing in rural areas are more likely to have unmet need for family planning as compared with women living in urban areas. The women who lack mass media exposure, who are not employed and who have fear of side effects for using contraceptives are more likely to have unmet need for family planning.

**Conclusions:**

Fear of side effects for using contraceptives has been identified as the major cause of unmet need for family planning in Pakistan. The Government of Pakistan has been putting a lot of efforts to convince people about the usefulness of population control programs. A huge media campaign has been launched to persuade people about the benefits of birth control. But the efforts of the government do not seem to be very much effective to clear the perception of people about side effects of contraceptive use. Hence, fear of side effects still remains one of the most important reason behind unmet need for family planning.

## Background

Unmet need for family planning (UMNFP) is defined as “the proportion of married women of reproductive age who are not using any method of family planning but would like to postpone the next pregnancy (unmet need for spacing), or who do not want any more children (unmet need for limiting)” [13, 14, 23, 31]; Woldemical and Beaujhot, 2011; [2, 11, 12].

Family planning services bring a large number of benefits to the welfare of women and the society. These services help in reducing the number of unwanted pregnancies and abortions. Family planning and reproductive health programs significantly contribute in improving child and maternal health by reducing fertility and can be useful to bring down maternal mortality rate in the developing countries [6, 25, 26, 28].

Despite significant implications of family planning program for economic development, unmet need for family planning (UMNFP) still exists particularly in developing countries of the world where 225 million women had unmet need for modern contraceptive methods during the year 2014 [24]. About 45% women, on average, had overall UMNFP in Asia, North Africa, and Eastern Europe (Albania, Armenia, Azerbaijan, Moldova and Ukraine) during the period of 2005–2011. In Western and Central Africa this figure was as high as 80% during the same period. Overall, UMNFP was 36% in Peru in the year 2008, 21% in Pakistan during 2012, 31% in Guyana in 2009, 32% in Tanzania during 2010, 31% in Ethiopia in 2011, 17% in Indonesia during 2007, 25% in Bangladesh during 2007, 22% in India during 2006 and 37% in Nepal in 2011 [32].

The prevalence of UMNFP is an important issue in developing countries [15]. Understanding causes and underlying factors of UMNFP can help to reduce the prevalence of UMNFP. Different factors such as limited choice and access of family planning methods, fear of side effects for using contraceptives (FSE), household’s income, number of living children, age of women, their region of residence (rural/urban), employment status of women, mass media exposure, husband’s education, religious or cultural constraints and poor quality of family planning services have been identified in the literature as important factors associated with UMNFP among women. Overall UMNFP is perceived to be higher among younger women, women living in rural areas and women who have lesser knowledge of methods and availability of contraceptives [18, 19, 30]. It is also found to be significantly lower among those women who belong to wealthier household and have a higher education [5, 9, 14, 18, 23, 27, 32]. Women with more number of children are less likely to have overall UMNFP than women with less number of children. In addition, urban women are less likely to have unmet need for contraceptives than rural women [4]. The findings of existing studies suggest that urban households in particular have smaller family size and often enjoy better access to family planning services than rural households, hence they have lower unmet need for family planning [8, 13, 21].

Pakistan is a developing country situated in the South Asian region. It is one among the top ten countries of the world with respect to population with a population growth rate of 2% [29]. The Government of Pakistan has been continuously struggling to bring down population growth by improving the availability of family planning services. It spends US $55 on each woman served, and US $17 on each couple-year protection [1]. Despite the efforts of the government, the acceptability and use of contraceptives in the country is low. Like many other developing countries of the world, UMNFP still exists in the country. According to Pakistan Demographic and Health Survey (PDHS) 2012–13, 56% of married women of reproductive age intended to use family planning services but only 35% of them were actually using these services. Thus the prevalence of overall unmet need for family planning was 21% in the country. The use of contraceptives was also different in different regions of the country. It was highest in Islamabad, the capital city which is one of the highest developed regions of the country. Whereas, it was lowest in Balochistan, the least developed province of the country. The prevalence of use of contraceptives among married women of Islamabad was 59% followed by 41% in Punjab, 34% in Gilgit Baltistan, 30% in Sindh, 28% in Khyber Pakhtunkhwa and 29% in Balochistan. The use of family planning methods among urban women was 45% against 31% among rural women of the country. The prevalence of overall UMNFP in the country was found to be 21% with 9.65% for spacing and 10.4% for limiting during the year 2012–13. This study is an attempt to investigate the determinants of UMNFP among married women in Pakistan.

## Methods

### Data source

The data used in our study is taken from PDHS 2012–13. The population of the survey consists of married women aged 15–49. A sample size of 14,000 women was selected out of which 6944 women belonged to urban areas and 7056 women were resident of rural areas. A number of 13,558 women were successfully interviewed. However, data for 560 respondents was reported in the survey with some missing observations. Thus comprehensive data of all variables used in our study was available for 12,998 women out of which 6941 belonged to rural and 6057 belonged to urban areas.

### Measurement

In order to investigate the determinants of UMNFP, the functional form of the model used in our study is as given below.

UMNFP = f (W.Age, W. Edu, ROR, NLC, WS, H. Edu, FSE, WES, EMM) (1).

where.

UMNFP = Unmet need for family planning has been used as dependent variable in our analysis. Overall unmet need was classified as having unmet need for family planning coded as 1 and not having unmet need for family planning coded as 0. Later on, unmet need was stratified as having unmet need for spacing coded as 1, having unmet need for limiting coded as 2 and not having any unmet need coded as 0.

W.Age = Women’s age has been classified into seven different age groups i.e. 15–19, 20–24, 25–29, 30–34, 35–39, 40–44 and 45–49.

W.Edu = Women’s education level has been classified into four different categories of no education, primary education, secondary education and higher education.

ROR = Region of residence is divided into two categories i.e. urban and rural. The geographical areas notified by the government authorities as urban areas have been considered as urban areas whereas the areas notified as rural areas have been treated as rural areas.

NLC = Number of living children of women.

WS = Wealth status of women’s household. The score of wealth index constructed by PDHS 2012–13 has been used to measure the status of women’s household. The index has been constructed by taking into account the different assets owned by household. Each household has been placed in five different quintiles with respect to their wealth index score. In the bottom quintile the poorest 20% households have been placed whereas the top quintile consists of the wealthiest 20% households. Thus the variable of wealth status of women’s household is a categorical variable with 5 different categories.

H.Edu = Husband’s education level has been classified into four categories of no education, primary, secondary and higher education.

FSE = Fear of side effects for using contraceptives. FSE reflects the women’s perception about the side effects of contraceptive use for their health. It is a categorical variable coded as 1 if the women have some FSE because they perceive that contraceptive use is not safe for their health. It is coded as 0 if they have no fear of side effects and consider that use of contraceptives is safe.

WES = Women’s employment status is divided into two categories of employed and not employed. Coded as 1, if employed and 0 otherwise.

EMM = Exposure to mass media. This variable provides information about the exposure of respondents about family planning methods. It is a categorical variable with two possible outcomes. If respondent has heard about family planning on the radio, seen anything about family planning on the television, read about family planning in a newspaper or magazine during the last few months then coded as 1 and 0 otherwise.

For our empirical analysis, we have used Binary Logistic and Multinomial Logistic regressions. The use of Binary Logistic regression is considered suitable if the dependent variable is a dichotomous variable. On the other hand, the use of Multinomial Logistic Regression is deemed to be appropriate when dependent variable is categorical variable with more than two possible values.

To find out the determinants of overall UMNFP, Binary Logistic regression analysis has been used because the variable was dichotomized as having unmet need or not having unmet need. Multinomial Logistic regression analysis has been used to find out the determinants of unmet need for family planning. Since unmet need for family planning was stratified as having unmet need for spacing coded as 1, having unmet need for limiting coded as 2 and not having any unmet need coded as 0.

## Results

According to the data reported in PDHS 2102–13, the prevalence of UMNFP in the country was found to be 21% with 9.65% for spacing and 10.4% for limiting during the year 2012–13. Socioeconomic characteristics of the respondent women are presented in Table 1.

**Table 1:** Socio-Economic Characteristics of Married Women

Socio-Economic Characteristics		Frequency	Percent (%)
Women’s age	15–19	546	4.2
	20–24	1950	15.0
	25–29	2599	20.0
	30–34	2327	17.9
	35–39	2197	16.9
	40–44	1729	13.3
	45–49	1650	12.7
Mean/Median/S.D age		30.87/30.00/1.21	
Region of Residence	Rural	6941	53.4
	Urban	6057	46.6
Women’s Education	No Education	7343	56.5
	Primary	1742	13.4
	Secondary	2301	17.7
	Higher	1612	12.4
Number of Living children of Women	0	1612	12.4
	1	1742	13.4
	2	1989	15.3
	3	1950	15.0
	4	1820	14.0
	5 or More	3885	29.9
Wealth status of Women’s Household	Poorest	2366	18.2
	Poorer	2470	19.0
	Middle	2469	19.0
	Richer	2535	19.5
	Richest	3158	24.3
Husband’s Education	No education	4147	31.9
	Primary	1729	13.3
	Secondary	4094	31.5
	Higher	3028	23.3
Fear of side effect for Contraceptive use	No Fear	3601	27.7
	Fear	9397	72.3
Women’s Employment status	Employed	2821	21.7
	Unemployed	10,178	78.3
Exposure of Mass Media	No	3327	25.6
	Yes	9671	74.4

Table 1 show that the mean age of the respondents is 30.87 years, standard deviation (S.D) is 1.21 and most of the respondents (69.8%) fall in the age-group of 20 years to 39 years. Similarly, the highest number of respondents (20%) lies in the age group of 25–29 and the lowest number of respondents (4.2%) fall in the age group of 15–19. 53.4% of the respondent women reside in rural areas as compared with 46.6% of urban women. Only 30.1% of the women have secondary or above secondary education whereas 56.5% women have no education and 13.4% of total women have primary education. 54.8% of the women’s husbands have secondary or above secondary education and 31.9% are uneducated. Similarly, 29.9% of the respondents have 5 or more than 5 children, 14% have 4 children, 15% have 3 children, 15.3% have 2 children and 13.4% have 1 child. 74.4% have exposure to mass media regarding family planning methods whereas only 25.6% of them have no such exposure. But 72.3% of the women have FSE. According to women’s employment status, 21.7% of the women are employed whereas 78.3% are unemployed.

The results of our Binary and Multinomial Logistic regression have been presented in Table 2. The results show that age of women is an important determinant of the likelihood of overall UMNFP. The reported odds ratios of the variable of women’s age indicate that the likelihood of overall UMNFP is highest among the women aged 20–24 and lowest among the women aged 45–49. It implies that with an increase in age, likelihood of overall UMNFP goes on to decrease. Women’s age has also been found to be significantly related with unmet need for spacing. The likelihood of unmet need for spacing goes on to decrease with an increase in age of women. The relationship of age with unmet need for limiting has been found to be non-linear. The odds ratios indicate that women belonging to second age group (20–24), third age group (25–29), fourth age group (30–34), fifth age group (35–39) and sixth age group (40–44) have higher unmet need for limiting than the women belonging to reference category of first age group (15–19). However, unmet need for limiting among women of seventh age group (45–49) is lower than the women of first age group (15–19). Unmet need for limiting has been found to be the highest among women of fifth age group (35–39). It is evident from our results that unmet need for spacing is highest among women of relatively younger age group (20–24) whereas unmet need for limiting is highest among middle aged women (35–39). Region of residence of women has also been found as an important determinant of overall UMNFP, unmet need for spacing and unmet need for limiting. In urban areas, likelihood of overall UMNFP, unmet need for spacing and unmet need for limiting has been found to be lower than rural areas. Results regarding the education of women show that with an increase in education, the likelihood of overall UMNFP, unmet need for spacing and unmet need for limiting goes on to decrease. Number of children of women shows a statistically significant relationship with overall UMNFP, unmet need for spacing and unmet need for limiting where women having four children have the lowest likelihood of facing overall UMNFP, unmet need for spacing and unmet need for limiting. Results of multinomial logistic regression show that likelihood of unmet need for limiting is highest among women having one child followed by women with two children. Women belonging to wealthier households are less likely to have overall UMNFP and unmet need for spacing. However, the likelihood of unmet need for limiting is highest among women belonging to second wealth quintile followed by women belonging to third and fourth wealth quintiles. Hence, it is found to be lowest either among richest or poorest women. With an increase in husband’s education, the likelihood of overall UMNFP, unmet need for spacing and unmet need for limiting has a tendency to fall. FSE is also a strong predictor of overall UMNFP, unmet need for spacing and unmet need for limiting. According to our data, 72.3% of the respondent women have FSE. Women having FSE are more likely to face overall UMNFP, unmet need for spacing and unmet need for limiting. Likelihood of overall UMNFP, unmet need for spacing and unmet need for limiting seems to be lower among employed women and among the women who have some mass media exposure.

**Table 2:** Determinants of unmet need for family planning among married women

Independent Variables		Binary LogisticRegression	Multinomial Logistic Regression	
		UMNFP	UMNFP For Spacing	UMNFP For Limiting
		Odds Ratio (*P*-value)	Odds Ratio (P-value)	Odds Ratio (P-value)
Women’s Age	15–19	Reference		
	20–24	1.701** (.023)	2.142*** (.002)	1.993* (.092)
	25–29	1.292* (.084)	1.580* (.071)	3.449* (.055)
	30–34	1.175* (.051)	1.390** (.017)	3.805** (.038)
	35–39	0.946** (.022)	0.374*** (.001)	4.299** (.024)
	40–44	0.454*** (.001)	0.043*** (.000)	2.212** (.018)
	45–49	0.177*** (.000)	0.003*** (.000)	0.881** (.044)
Region of Residence	Rural	Reference		
	Urban	0.860* (.098)	0.860* (.068)	0.743** (.022)
Women’s Education	No Education	Reference		
	Primary	1.556* (.072)	1.637** (.041)	1.572* (.086)
	Secondary	1.273** (.050)	1.215* (.079)	1.304* (.062)
	Higher	1.239** (.011)	1.202** (. 040)	1.263** (.026)
Number of Living Children of Women	0	Reference		
	1	0.637*** (.002)	0.180*** (.001)	1.532*** (.000)
	2	0.441*** (.003)	0.137*** (.000)	1.067** (.043)
	3	0.405** (.024)	0.094*** (.000)	0.945** (.020)
	4	0.306** (.042)	0.072*** (.000)	0.433* (.089)
	5 or More	0.394* (.057)	0.095*** (.000)	0.583** (.048)
Wealth Status of Women’s Household	Poorest	Reference		
	Poorer	0.950** (.043)	0.770** (.012)	1.043* (.085)
	Middle	0.939* (.063)	0.729* (.079)	1.034* (.052)
	Richer	0.901* (.080)	0.712* (.092)	1.009** (.026)
	Richest	0.861** (.047)	0.627** (.050)	0.964** (.037)
Husband’s Education	No education	Reference		
	Primary	1.242* (.056)	1.139** (.010)	1.271* (.054)
	Secondary	1.056* (.066)	0.980* (.085)	1.064* (.059)
	Higher	0.950* (.078)	0.937* (.081)	0.927* (.079)
Fear of Side Effect for Contraceptive Use	No Fear	Reference		
	Fear	2.327*** (.000)	2.173*** (.000)	2.372* (.000)
Women’s Employment Status	Unemployed	Reference		
	Employed	0.986* (.073)	0.867** (.031)	1.053* (.095)
Exposure to Mass Media	No	Reference		
	Yes	0.815** (.011)	0.964** (.045)	0.851* (.087)

Note: * Significant at 10%; ** Significant at 5%; *** Significant at 1%

## Discussion

Women’s age, their region of residence (rural/urban), education, number of living children, wealth status of their households, education of their husbands, FSE, their employment status and exposure to mass media have been identified as important determinants of unmet need for family planning among married women of Pakistan. It is to be noted that likelihood of overall UMNFP and unmet need for spacing is highest among younger women aged 20–24. However, the likelihood of unmet need for limiting is highest among women in the middle age group (35–39). Demand for limiting can be expected to be higher among women in the middle age group (35–39). They might have already attained their desired number of children and hence would like to use contraceptives for limiting. On the other hand, demand for spacing can be expected to be higher among younger women who want to postpone their next pregnancy. Our results confirm the findings of the earlier studies [3, 9, 11, 18, 31, 33]. The higher likelihood of overall UMNFP, unmet need for spacing and unmet need for limiting in rural areas than that of urban areas is an indication of easy availability of family planning services in urban areas. Existing studies conducted in different countries and regions of the world also suggest that the likelihood of unmet need for family planning has been found to be higher in rural areas as compared to urban areas in case of Turkey [10], Rwanda [17], India [3, 9], Nepal, [20] and Ethiopia [16].

Educated women are less likely to have overall UMNFP, unmet need for spacing and unmet need for limiting than uneducated women. The women with higher education have the lowest overall UMNFP, unmet need for spacing and unmet need for limiting. It indicates that educated women are more likely to avail family planning services because they are well informed. Similarly, education of women makes them more empowered in decision making regarding contraceptive use [22]. These results support the findings of previous studies of Devis et al., [9], Klijzing [14], Ojakaa [18] and Choudhary et al. [7].

According to our results, the likelihood of overall UMNFP, unmet need for spacing and unmet need for limiting decreases with increase in number of children. Such likelihood goes on to decreases till the birth of fourth child. It is the lowest among the women having four children. However women with five or more number of children have slightly higher UMNFP, unmet need for spacing and unmet need for limiting than women with four number of children. It indicates that with an increase in number of children, women and other family members who can possibly be involved in fertility decisions may become more convinced regarding the usefulness of family planning methods. However, this relationship is non-linear.

The women belonging to wealthier households are likely to have lower overall UMNFP, unmet need for spacing and unmet need for limiting than the women of poorer households. It is so because wealthier households can have better access to modern contraceptives as compared to poorer households. Apart from women’s own education, their husband’s education can also reduce the likelihood of overall UMNFP, unmet need for spacing and unmet need for limiting because fertility decisions in any household can be a joint decision made by women and their husbands. Educated individuals may be well informed and can have better access to family planning services. Fear of side effects can increase the likelihood of unmet need for family planning. This indicates that people who intend to use contraceptives may not opt to do so simply because they have FSE. The Government of Pakistan has been putting a lot of efforts to convince people about the usefulness of population control programs. A huge media campaign has been launched where the messages of celebrities and notable personalities of the society are communicated to people to persuade them about the benefits of birth control. Electronic as well as print media is being used for this purpose. But the effort of the government does not seem to be very much effective to clear the perception of people about side effects of contraceptive use. Hence, FSE still remains one of the most important reason behind over all UMNFP. Employment of women against some paid job increase their opportunity cost of bearing and rearing of a child. Their responsibility of bearing and rearing of a child can reduce their time devoted to paid work and hence they will have to forego their income. Thus employed women are expected to be more concerned regarding their family size. As a result of it, a decreased likelihood of unmet need for family planning is expected among employed women. Women’s exposure to mass media can help to reduce the likelihood on overall UMNFP, unmet need for spacing and unmet need for limiting. Such exposure can provide them information regarding the availability and usefulness of different family planning methods. Thus an effective media campaign can be useful to reduce UMNFP. It can help people to limit their family size according to their desire.

The reported results are based upon the data of the respondent women aged 15–49. However, family planning decisions can be heavily influenced by the socioeconomic characteristics, views and perceptions of men about the family planning services. This can be particularly true in a male-dominated society like Pakistan. Future research can be helpful to explore the significance of these factors for the prevalence of unmet need for family planning. Moreover, the causes of the regional differences of overall UMNFP need to be explored.

## Conclusions

The Government of Pakistan has been continuously struggling to bring down population growth in the country because the size and growth rate of population can have enormous implications for economic development. However, the prevalence of overall UMNFP can hamper these efforts. Thus exploring the determinants of unmet need for family planning can be helpful to cope with the issue by formulating appropriate policies. This study has identified some important determinants of overall unmet need for family planning among married women in Pakistan. In the light of our empirical results, we suggest that education and particularly female education can be used as an effective tool to reduce overall unmet need for family planning. Women’s access to education must be ensured. This would be effective not only for increasing their awareness about reproductive health and modern contraceptive use but also for empowering them in the family. As a result, they will have an increasing role in fertility decision making and would have more autonomy to use contraceptives if they wish to limit their family size. Moreover, an effective media campaign needs to be launched to create awareness among people about the availability and usefulness of family planning methods for population control. Such campaign may also be helpful to remove people’s FSE. The provision of better quality contraceptives can also be helpful to remove such fear of side effects. Creation of employment opportunities is another tool which can be used to reduce overall unmet need for family planning in the country. Employed women can be more empowered and hence they can be in better position to take decisions about the use of family planning methods. Social and cultural barriers for employment of women need to be abolished by the government through effective public policy, mass spread of education and effective media campaign. Region of residence has been explored as an important determinant of overall UMNFP. Rural areas not only lack in facilities of family planning services but also have certain social taboos due to which the likelihood of overall UMNFP has been found to be higher in rural areas than that of urban areas. Community leaders and religious scholars need to be engaged to convince people about the usefulness of family planning program. Hence, rural areas should be the particular focus of family planning program in the country.

## Data Availability

We have used the secondary data of PDHS 2012–13. Available at: https://www.nips.org.pk/study_detail.php?detail=MTgw

## Abbreviations

FSE: Fear of Side Effect for Using Contraceptives
PDHS: Pakistan Demographic and Health Survey
UMNFP: Unmet Need for Family Planning
