# Correction to: Socio-demographic determinants of unmet need for family planning among married women in Pakistan

**DOI:** 10.1186/s12889-021-10264-3

**Published:** 2021-01-25

**Authors:** Muhammad Farhan Asif, Zahid Pervaiz

**Affiliations:** Department of Economics National College of Business Administration and Economics 40-E/1 Gulberg III, Lahore, Pakistan

**Correction to: BMC Public Health (2019) 19:1226**

**http://orcid.org/10.1186/s12889-019-7487-5**

It was highlighted that in the original article [1] the authors reported the descriptive statistics without excluding the missing values. As the data of FSE was available for 3773 respondents the sample size was actually restricted to 3770 (for which the data of all variables was available). Furthermore, in the variable of Mass Media Exposure responses of “Yes” had been erroneously labelled as “No” and vice versa in word file. This Correction article shows the correct files, Table 1, and the correct sections of the article.

**Table 1:** Socio-Economic Characteristics of Married Women

Socio-Economic Characteristics		Frequency	Percent (%)
**Women’s age**	15–19	110	2.9
	20–24	456	12.1
	25–29	569	15.1
	30–34	608	16.1
	35–39	589	15.6
	40–44	642	17.0
	45–49	796	21.1
**Region of Residence**	Rural	2179	57.8
	Urban	1591	42.2
**Women’s Education**	No Education	2497	66.2
	Primary	451	13.4
	Secondary	527	17.7
	Higher	295	12.4
**Number of Living children of Women**	0	40	12.4
	1	432	13.4
	2	527	15.3
	3	609	15.0
	4	596	14.0
	5 or More	1566	29.9
**Wealth status of Women’s Household**	Poorest	827	21.9
	Poorer	804	21.3
	Middle	744	19.7
	Richer	663	17.6
	Richest	732	19.4
**Husband’s Education**	No education	1393	36.9
	Primary	504	13.4
	Secondary	1151	30.5
	Higher	722	19.2
**Fear of side effect for Contraceptive use**	No Fear	3219	85.4
	Fear	551	14.6
**Women’s Employment status**	Employed	800	21.2
	Unemployed	2970	78.8
**Exposure of Mass Media**	No	2966	78.7
	Yes	804	21.3

## Data source

The data used in our study is taken from PDHS 2012–13, conducted by the National Institute of Population Studies (2013). The population of the survey consists of married women aged 15–49. A sample size of 14,000 women was selected out of which 6944 women belonged to urban areas and 7056 women were resident of rural areas. A number of 13,558 women were successfully interviewed. However, data for 9788 respondents was reported in the survey with some missing observations. Thus comprehensive data of all variables used in our study was available for 3770 women out of which 2179 belonged to rural and 1591 belonged to urban areas.

## Results

According to the data reported in PDHS 2102–13, the prevalence of UMNFP in the country was found to be 21% with 9.65% for spacing and 10.4% for limiting during the year 2012–13. Socioeconomic characteristics of the respondent women are presented in Table 1.

Table 1 show that most of the respondents (58.9%) fall in the age-group of 20 years to 39 years. Similarly, the highest number of respondents (21.1%) lies in the age group of 45–49 and the lowest number of respondents (2.9%) fall in the age group of 15–19. 57.8% of the respondent women reside in rural areas as compared with 42.2% of urban women. Only 30.1% of the women have secondary or above secondary education whereas 66.2% women have no education and 13.4% of total women have primary education. 49.7% of the women’s husbands have secondary or above secondary education and 36.9% are uneducated. Similarly, 29.9% of the respondents have 5 or more than 5 children, 14% have 4 children, 15% have 3 children, 15.3% have 2 children and 13.4% have 1 child. 21.3% have exposure to mass media regarding family planning methods whereas only 78.7% of them have no such exposure. But 85.4% of the women have FSE. According to women’s employment status, 21.2% of the women are employed whereas 78.8% are unemployed.
